# Examining measurement discrepancies in adolescent screen media activity with insights from the ABCD study

**DOI:** 10.1038/s44184-025-00131-z

**Published:** 2025-05-10

**Authors:** Yihong Zhao, Xuewei Han, Kara S. Bagot, Susan F. Tapert, Marc N. Potenza, Martin P. Paulus

**Affiliations:** 1 Columbia University School of Nursing, New York, NY USA; 2 Department of Psychiatry and Biobehavioral Sciences, University of California Los Angeles, Los Angeles, CA USA; 3 Department of Psychiatry, University of California San Diego, San Diego, CA USA; 4 Department of Psychiatry, Child Study Center, Department of Neuroscience, Yale University School of Medicine, New Haven, CT USA; 5 Connecticut Mental Health Center, New Haven, CT USA; 6 Connecticut Council on Problem Gambling, Wethersfield, CT USA; 7 Wu Tsai Institute, Yale University, New Haven, CT USA; 8 Laureate Institute for Brain Research, Tulsa, OK USA; 9 University of California San Diego, Department of Psychiatry, Seattle, WA USA

**Keywords:** Human behaviour, Psychology, Risk factors, Human behaviour

## Abstract

Concerns about the accuracy of self-reported screen time persist due to discrepancies with objective measures. This study compared passive smartphone tracking via the “Effortless Assessment of Risk States’’ (EARS) app with self-reported screen time from 495 adolescents. Based on self-reports, 94.26% of social media use occurred on smartphones. EARS-recorded social media use was higher (1.64 ± 1.93 h) than past-year self-report (1.44 ± 1.97 h; *p* = 0.037) but similar to post-sensing self-report (1.63 ± 1.93 h; *p* = 0.835). Higher picture vocabulary scores were associated with lower odds of under-reporting social media use (OR = 0.96, 95% CI: 0.93–0.99). Both self-reported (*β* = 0.06, 95% CI: 0.01–0.11) and EARS (*β* = 0.07, 95% CI: 0.03–0.12) measures correlated with externalizing symptoms. They were also correlated with social media addiction (self-reported:*β* = 0.15, 95% CI: 0.10–0.20; EARS:*β* = 0.06, 95% CI: 0.01–0.11). However, past-year self-report uniquely correlated with internalizing symptoms (*β* = 0.05, 95% CI: 0.01–0.09) and video game addiction (*β* = 0.05, 95% CI: 0.01–0.10). These findings highlight the value of integrating self-report and objective measures in screen media use research.

## Introduction

Screen media activity (SMA) and its relationship with health, particularly among adolescents, is a growing concern^1–3^. SMA has been linked with mental health^4,5^, cognitive^6–8^, and brain^9–11^ measures with mixed findings, leading to ongoing discussions about its effects on various health outcomes^12^. One challenging issue in this field involves potential discrepancies between self-reported and objectively measured SMA time, raising concerns about measurement bias and reliability of self-reported SMA estimates^13^. Much research on SMA relies on self-reported retrospective recall, yet mounting data suggest that individuals may lack insight into their actual SMA patterns^14–18^. As with self-report studies generally, self-reported SMA is vulnerable to multiple types of biases involving recall, heuristic distortions, and social desirability^2,3,13,19,20^, particularly in adolescents with potentially limited introspective accuracy to estimate usage durations across multiple platforms^21^. Cognitive biases, such as the peak-end rule, where individuals recall extreme or final moments of an experience rather than cumulative exposure, may further distort self-reports^22^. In addition, developmental factors, including executive function maturation and attentional shifts^23^, may generate inconsistencies in perceived versus actual screen engagement. If not properly considered, such biases could obscure associations between SMA and health indicators^24,25^.

Beyond cognitive factors, discrepancies in self-reported SMA estimates also vary by device types and demographic characteristics^26^. For example, parents tend to overestimate their children’s texting and video chatting while underestimating multiplayer gaming and social media use, compared to adolescent self-reports. Parents generally report higher overall screen time, whereas adolescents report greater engagement in interactive and peer-driven activities, such as multiplayer gaming and social networking. Moreover, larger discrepancies in total recreational screen time have been observed in older, Black, and Latino/Hispanic adolescents, as well as among youth from unmarried households^26^.

To address these challenges, researchers have suggested incorporating objective measures, which may provide more accurate and reliable data than self-reports^27^. Passive-sensing technologies offer new possibilities for capturing real-time SMA behaviors, thus reducing biases associated with recall errors and subjective estimation. Studies have utilized applications, such as Apple screen time, the moment app, android-native digital wellbeing, and effortless assessment of risk states (EARS) to collect objective SMA measures^28–31^. While objective measures have demonstrated moderate positive correlations with self-reported SMA^13^, emerging evidence suggests that self-reports and objective measures may capture related yet distinct constructs rather than interchangeable measures of the same underlying media consumption behaviors^32,33^. If discrepancies were non-random, then such discrepancies may reflect cognitive biases, self-monitoring limitations, and/or social desirability effects that may influence their perceived SMA and technology use^32^.

To interpret potential discrepancies within a broader developmental and social cognitive context, we can draw on Bronfenbrenner’s ecological systems theory^34^, which we have discussed previously^2^, considering how SMA operates within nested environmental and cognitive contexts. This model accounts for individual, familial, peer, and societal factors that may shape SMA’s impact on psychological development. Self-reporting discrepancies may vary by age, socioeconomic status, and cultural background, and measurement biases may reflect both individual cognitive limitations and broader environmental influences^26^. Additionally, social cognitive theory^35^ suggests that individuals’ perceptions of their own behaviors are shaped by cognitive heuristics, observational learning, and social norms, which may lead to biases in self-reported SMA. In particular, adolescents are developing self-awareness and self-monitoring skills^36,37^, which may influence how accurately individuals report their own SMA. Given that self-awareness has been positively associated with executive functioning^37^, individual differences in working memory, inhibitory control, and cognitive flexibility may relate to recall bias in self-reported SMA.

In this investigation, we analyzed the self-reported and objectively logged SMA from the adolescent brain cognitive development (ABCD) Study^38^, where the EARS App^28^ was used to collect objectively logged SMA^27,39^. Here, we aimed to assess: 1) the extent to which differences existed between self-reported screentime and EARS-logged app usage, 2) whether participants’ cognitive measures were related to such differences, and 3) whether self-reported and objective screentime were differentially associated with behavioral problems and problematic usage of digital technologies.

## Methods

### Participants

Individuals participated in the ABCD study^38^. Release 5.1 data included 4754 participants at Year-4 follow-up. Of these, 1483 participants (753 girls) agreed to install the EARS smartphone app^28^, which passively collected app usage data for 3 weeks. However, EARS-logged data were only available for 495 Android users, as Apple blocked app-scrapping programs at data-collection time. Therefore, analyses focused on 495 participants with the EARS-logged data.

### SMA usage time

Subjective SMA was assessed by youth self-reported time involved in screen activities (TV/video, video games, text, social media, etc.) on a typical day over the past 12 months from the ABCD Screen Time Questionnaire^40^. Post-sensing survey data about SMA during the three-week sensing period were also analyzed. Analyses focused on total daily hours and hours spent on social media and video gaming.

Objective usage time was extracted from the raw EARS data. Total EARS-logged time was calculated as the sum of the foreground app usage times across all apps averaged over days with at least one upload during the tracking period. System operational app usage times were excluded. Social media and game app usage were summarized by adding all app usage times on social media and game apps as defined by Google Play, respectively. Snapchat was labeled as a social media app in this study due to its multifunctionality, despite being listed as a communication app by Google Play. Some entertainment apps (e.g., Google Play Games, Xbox, PlayStation App, and Steam) were redefined as game apps. A list of key social media and game apps used by participants is provided in Supplementary Table 1. Detailed definition of self-reported SMA usage time can be found in Supplementary Table 2. Proportion of time spent on each SMA category was calculated as the time spent on that category over total time across all SMA on a typical day.

### Behavioral problems

Youth behavioral problems (*n* = 492) were assessed using the parent-report child behavior checklist^41^ subscales in the domains of internalizing problems (i.e., withdrawn/depressed, anxiety/depressed, somatic complaints) and externalizing problems (i.e., rule-breaking behavior, aggressive behavior). Higher scores reflect more behavioral problems.

### Cognitive functions

At the Year-4 follow-up, four cognitive subscales from the NIH toolbox (picture vocabulary, reading, picture sequencing, and list sorting) had minimal missing data (*n* ≤ 20) and were selected as the primary cognitive measures^42^. The other cognitive measures (e.g., card sorting, flanker test, and pattern comparison) had substantial missingness and thus were excluded from analyses. NIH toolbox cognitive measures are widely used to investigate neurocognitive maturation in youth^43^. Higher scores reflect better performance. We hypothesized that individuals with higher cognitive scores would have lower reporting bias.

### Problematic technology usage

The relationships between objective and subjective screen usage durations and compulsive/problematic SMA were assessed using data from the social media addiction questionnaire (SMAQ) and videogame addiction questionnaire (VGAQ). These scales, adapted from the Bergen Facebook Addiction Scale^44^, demonstrate good psychometric properties^27^. In this sample, the internal reliability, as measured by McDonald’s ω, was 0.89 for SMAQ and 0.90 for VGAQ, consistent with previously reported values^27^. They measure addiction severity related to social media and video games by evaluating respondents’ frequency of specific behaviors or feelings about their technology use. Higher totals reflect greater technology-related concerns.

### Statistical analysis

Pearson correlations and paired two-sample *t* tests were conducted to assess the strength and significance of associations between self-reported and objectively measured app usage times. Logistic regression models were used to evaluate the relationship between reporting bias in SMA and cognitive performance. Linear mixed models, with family as a random effect, were used to examine associations between SMA (both subjective and objective) and behavioral problems. Standardized estimates were reported to facilitate cross-measure comparisons. Basic demographic variables (Table 1) were considered as potential covariates. Missing data on family income, parental education, and parental marital status were imputed using the last-observation-carried-forward approach, as these variables are relatively stable over time. After imputation, seven participants still had missing data on family income, and these cases were excluded from relevant analyses. No imputations were performed for missing cognitive function or self-reported SMA data. In sensitivity analysis, we included pubertal development stage as a covariate. Pubertal development stage was derived primarily from parent reports, but when missing, youth self-reports were used. Three participants had missing data on pubertal development stage. All analyses were conducted in *R* using the lme4 package. Two-sided tests were used, with a significance level of 0.05.

**Table 1:** Demographic characteristics of ABCD participants with and without EARS data in ABCD Data Release 5.1^a^

	Included (*n* = 495)	Excluded (*n* = 4259)	*p* value
Age (years)	14.01 (0.70)	14.09 (0.68)	0.019
Sex assigned at birth			
Male	288 (58.18%)	2201 (51.68%)	0.007
Female	207 (41.82%)	2058 (48.32%)	
Race ethnicity			
White	281 (56.77%)	2401 (56.37%)	0.943
Black	54 (10.91%)	455 (10.68%)	
Hispanic	97 (19.60%)	882 (20.71%)	
Other	63 (12.73%)	521 (12.23%)	
Family annual income, US $^b^			
$$\ge$$100,000	202 (40.81%)	2217 (52.05%)	<0.001
50,000–100,000	154 (31.11%)	1030 (24.18%)	
<50,000	132 (26.67%)	962 (22.59%)	
Missing	7 (1.41%)	50 (1.17%)	
Parental education^b^			
Post-graduate degree	146 (29.49%)	1651 (38.76%)	<0.001
Bachelor	145 (29.29%)	1128 (26.49%)	
Some college	132 (26.67%)	883 (20.73%)	
High school or below	72 (14.55%)	596 (13.99%)	
Missing	0 (0%)	1 (0.02%)	
Parental marital status^b^			
Married	338 (68.28%)	2964 (69.59%)	0.584
Not married	157 (31.71%)	1295 (30.41%)	

^a^Mean (SD) for continuous variables, *n* (%) for categorical variables. EARS refers to the logged data collected by the EARS app.

^b^Variables were imputed using last observation carried forward approach if they were missing at Year-4 follow-up.

### Ethics

IRB approval and informed written consent was obtained from participating ABCD sites during data collection. The current analyses involved deidentified data and were exempted by the Columbia University IRB. Thus, the study is in accordance with the principles of the Declaration of Helsinki.

## Results

### Most participants reported no change in screen usage relative to EARS app

Basic demographic information for the subset of 495 participants with EARS-logged data at the Year-4 follow-up is summarized in Table 1. Compared to ABCD participants with 4-year follow-up data (*N* = 4259), the EARS sample (*n* = 495) had a higher proportion of boys (58.18% vs. 51.68%), slightly younger ages (14.01 vs. 14.09 years), more parents with education levels up to some college (42.03% vs. 35.78%), and more families earning ≤$100,000 annually (50.91% vs. 40.70%). No differences were found in parental marital status or race/ethnicity between the study sample and the full ABCD cohort.

During the three-week EARS-sensing period, participants averaged 20.71 ± 4.14 active usage days, interacted with 20.20 ± 6.81 different apps per day, and cumulatively used 54.74 ± 21.30 unique apps. The top five social media apps and the top five game apps are presented in Supplementary Table 3. Of 397 participants with post-EARS survey data, most participants expressed willingness to continue using the EARS app for a longer period (*n* = 283, 71.28%) and answer daily questions (*n* = 299, 75.31%). A small number (*n* = 16, 4.03%) reported someone else using their phones for at least 25% of time during the sensing period. Importantly, most (*n* = 315, 79.35%) reported no change in screen usage during the sensing period, with small portions reporting decreased (*n* = 37, 9.32%) or increased (*n* = 45, 11.34%) usage.

### Objective and subjective social media use

**Fig. 1:**
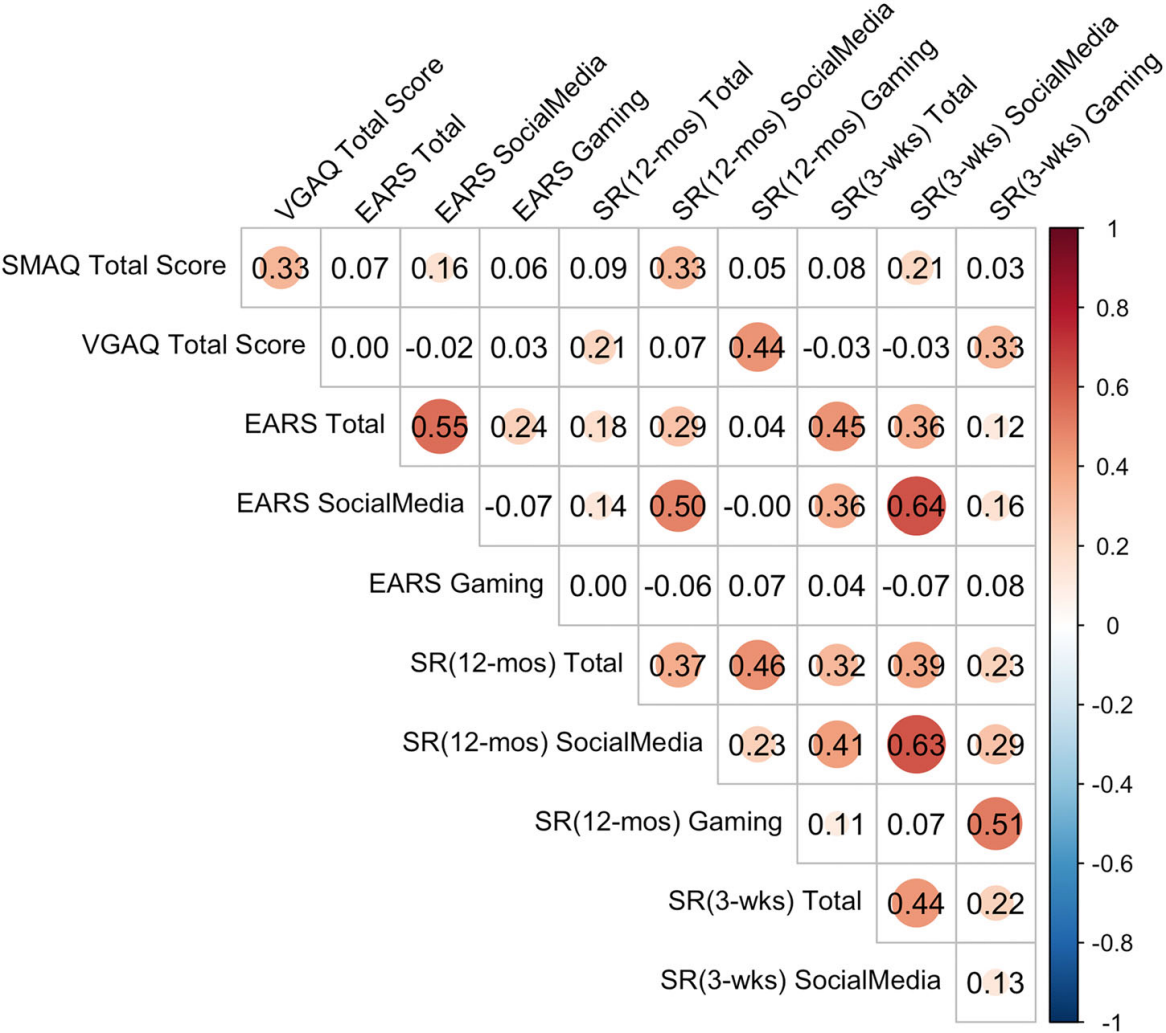
Pairwise correlation among key SMA measures, including self-reported screen time, EARS-related measures, and addiction scores among all available data. The number in each cell stands for the correlation coefficient between two variables. Significant correlations are highlighted with circles, where the size and color indicated the strength and direction of the association.

Consistent with previous research^39^, EARS-logged usage was moderately correlated with self-reported screentime during the sensing period (*r* = 0.45, 95% CI 0.36–0.52) and prior 12 months (*r* = 0.18, 95% CI: 0.09–0.26). Correlations were higher for social media time during the sensing period (*r* = 0.64, 95% CI: 0.57–0.69) than the prior 12 months (*r* = 0.50, 95% CI: 0.43–0.56). EARS-recorded gaming time was not correlated with self-reported gaming during the sensing period (*r* = 0.08, 95% CI: −0.02, 0.18) or prior 12 months (*r* = 0.07, 95% CI: −0.02, 0.16). Pairwise correlations among subjective and objective SMA categories are shown in Fig. 1.

Mean EARS total app usage (4.89 h ± 2.87) was similar to self-reported total screen time during the three-week sensing period (4.73 h ± 3.81; *p* = 0.373) and over the prior 12 months (5.27 h ± 3.02; *p* = 0.053), suggesting general agreement in overall usage across measurement types (Table 2). However, divergences emerged in specific categories. EARS recorded higher social media use (1.64 h ± 1.93) compared to past-year self-reported time (1.44 h ± 1.97; *p* = 0.037), and EARS gaming time (0.52 h ± 0.73) was significantly lower than self-reports over the sensing period (2.48 h ± 2.99) and prior year (3.40 h ± 3.39) with *p* values <0.001 (Table 2).

**Table 2:** Difference in usage time between objective and subjective measures^a^

Usage time (hours)	Sample Size	EARS^b^	SR^c^(3-weeks)	SR^c^(12-months)
Total				
Mean (SD)	*n* = 376	4.89 (2.87)	4.73 (3.81)	5.27 (3.02)
Median [Min, Max]		4.62 [0.04, 17.06]	3.57 [0, 24.00]	4.73 [0, 23.21]
Social Media				
Mean (SD)	*n* = 395	1.64 (1.93)	1.63 (1.93)	1.44 (1.97) *
Median [Min, Max]		0.93 [0, 8.97]	1.00 [0, 17.86]	0.86 [0, 13.50]
Gaming				
Mean (SD)	*n* = 395	0.52 (0.73)	2.48 (2.99) ***	3.40 (3.39) ***
Median [Min, Max]		0.28 [0, 6.97]	1.50 [0, 24.00]	2.43 [0, 23.93]

^a^*** means *p* value <0.001; ** means *p* value <0.01; * means *p* value <0.05. *P* values were calculated based on paired two-sample *t* test.

^b^EARS= logged data by the EARS app.

^c^SR=self-report; SR (3-weeks) means post-sensing self-report data; SR (12-months) means past-year self-report data.

We note that based on past-year self-report data, 94.26% ( ± 18.37%) of time spent on social media took place on smartphones, while for gaming, only 33.47% ( ± 37.95%) occurred on smartphones.

### Under-reporting of social media time and its relationship with picture vocabulary

**Fig. 2:**
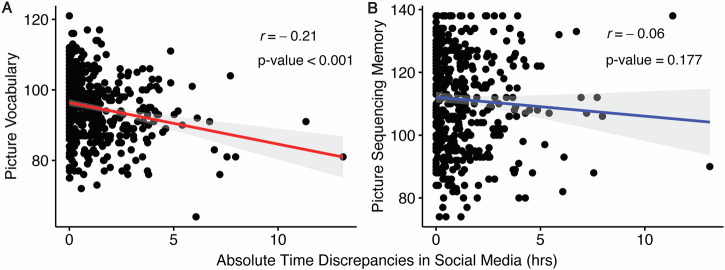
Scatterplot of absolute differences between objective and subjective social media usage against cognitive measurements. Absolute time discrepancy in objective and subjective social media usage was significantly related to lower picture vocabulary score (**A**) but not picture sequencing memory score (**B**). Here,subjective social media usage was based on youth self-report over past 12 months.

Participants were categorized into over-reporting, under-reporting, and accurate-reporting youth based on agreement between the EARS-logged and self-reported social media usage in the prior 12 months. Over-reporting youth were those whose self-reported usage exceeded EARS-logged time by at least 30 min, and under-reporting youth had self-reported usage at least 30 min less than the EARS-logged time. In this sample, 22.06% (*n* = 109) of participants over-reported and 30.97% (*n* = 153) under-reported. Pairwise correlation plots for participants classified as accurate reporters and those with reporting bias (i.e., over-reporters and under-reporters) are presented in Supplementary Fig. 1. Notably, youth who under- or over-estimated their social media usage exhibited lower Picture vocabulary scores compared to accurate-reporting youth, with both over-reporting and under-reporting youth demonstrating lower scores than accurate-reporting respondents (94.16 ± 8.73 and 93.03 ± 8.61 vs. 96.74 ± 8.76). Importantly, when controlling for age, sex, race, family income, parental education levels, and parental marital status, higher Picture vocabulary scores were associated with lower odds of under-reporting (Odds ratio = 0.96, 95% CI:0.93–0.99). However, no association was observed with over-reporting (Odds ratio = 0.98, 95% CI:0.95–1.01), possibly due to the smaller sample size in the over-reporting group (Table 3a, b and Fig. 2). Here, survey time was recorded in 15-min increments. Therefore, a 30-min cutoff was used to represent a deviation of two measurement units. As part of the sensitivity analysis, we found that pubertal development stage was not associated with reporting bias and did not confound the associations between reporting bias and the two cognitive measures (Supplementary Table 7a, b). With and without controlling for pubertal development stages, reporting bias was not associated with picture sequencing memory (Supplementary Tables 4a, b), list sorting working memory test (Supplementary Table 5a, b), and oral reading recognition test (Supplementary Table 6a, b).

**Table 3:** a Multinomial logistic regression models testing associations between NIH toolbox picture vocabulary and accurate- and over-reporting of social media use; b Multinomial logistic regression models testing associations between NIH toolbox picture vocabulary and accurate- and under-reporting of social media use

a				
	Cutoff = 0.5 h		Cutoff = 1h	
	Accurate: 222; Over: 106		Accurate: 290; Over: 69	
	OR [95% CI]	*p* value	OR [95% CI]	*p* value
Picture vocabulary	0.98 [0.95–1.01]	0.233	0.98 [0.95–1.02]	0.286
Age (yrs)	1.24 [0.87–1.77]	0.241	1.20 [0.80–1.79]	0.376
Sex at birth (Ref: male)				
Female	1.09 [0.66–1.80]	0.724	1.09 [0.62–1.91]	0.755
Race ethnicity (Ref: white)				
Black	3.31 [1.23–8.90]	0.018	2.98 [1.13–7.82]	0.027
Hispanic	0.87 [0.43–1.78]	0.706	1.31 [0.59–2.89]	0.502
Other	0.63 [0.29–1.38]	0.249	0.81 [0.33–1.97]	0.636
Family income (US$) (Ref: [>100k])				
[50–100k]	0.75 [0.40–1.40]	0.363	0.68 [0.33–1.43]	0.315
[<50k]	0.76 [0.32–1.79]	0.531	0.67 [0.26–1.74]	0.407
Parental education (Ref: post graduate degree)				
Bachelor	1.13 [0.60–2.14]	0.703	0.71 [0.34–1.49]	0.361
Some college	1.32 [0.61–2.82]	0.481	1.07 [0.46–2.46]	0.876
High school or below	1.46 [0.55–3.86]	0.449	1.03 [0.35–3.01]	0.960
Parental marital (Ref: married)				
Not married	2.27 [1.26–4.07]	0.006	1.66 [0.87–3.19]	0.126

b				
	Cutoff = 0.5 h		Cutoff = 1 h	
	Accurate: 222; under: 145		Accurate: 290; under: 114	
	OR [95% CI]	*p* value	OR [95% CI]	*p* value
Picture vocabulary	0.96 [0.93–0.99]	0.010	0.96 [0.93–0.99]	0.007
Age (yrs)	1.42 [1.03–1.96]	0.034	1.43 [1.03–1.99]	0.035
Sex at birth (Ref: male)				
Female	1.17 [0.75–1.83]	0.494	1.12 [0.71–1.78]	0.624
Race ethnicity (Ref: white)				
Black	2.04 [0.78–5.34]	0.148	1.33 [0.55–3.24]	0.523
Hispanic	1.22 [0.67–2.22]	0.524	1.54 [0.84–2.83]	0.159
Other	0.74 [0.37–1.48]	0.392	0.71 [0.33–1.52]	0.376
Family Income (US$) (Ref: [>100k])				
[50–100k]	0.69 [0.38–1.23]	0.205	0.91 [0.50–1.66]	0.763
[<50k]	1.00 [0.47–2.13]	1.000	1.05 [0.48–2.28]	0.901
Parental education (Ref: post graduate degree)				
Bachelor	1.14 [0.63–.07]	0.658	1.12 [0.59–2.10]	0.728
Some college	1.71 [0.86–3.41]	0.128	1.36 [0.66–2.80]	0.400
High school or below	1.44 [0.58–3.54]	0.428	1.44 [0.59–3.56]	0.426
Parental marital (Ref: married)				
Not married	1.55 [0.90–2.68]	0.114	1.14 [0.66–1.98]	0.635

### Social media use relationships with behavioral problems and problematic technology usage

**Fig. 3:**
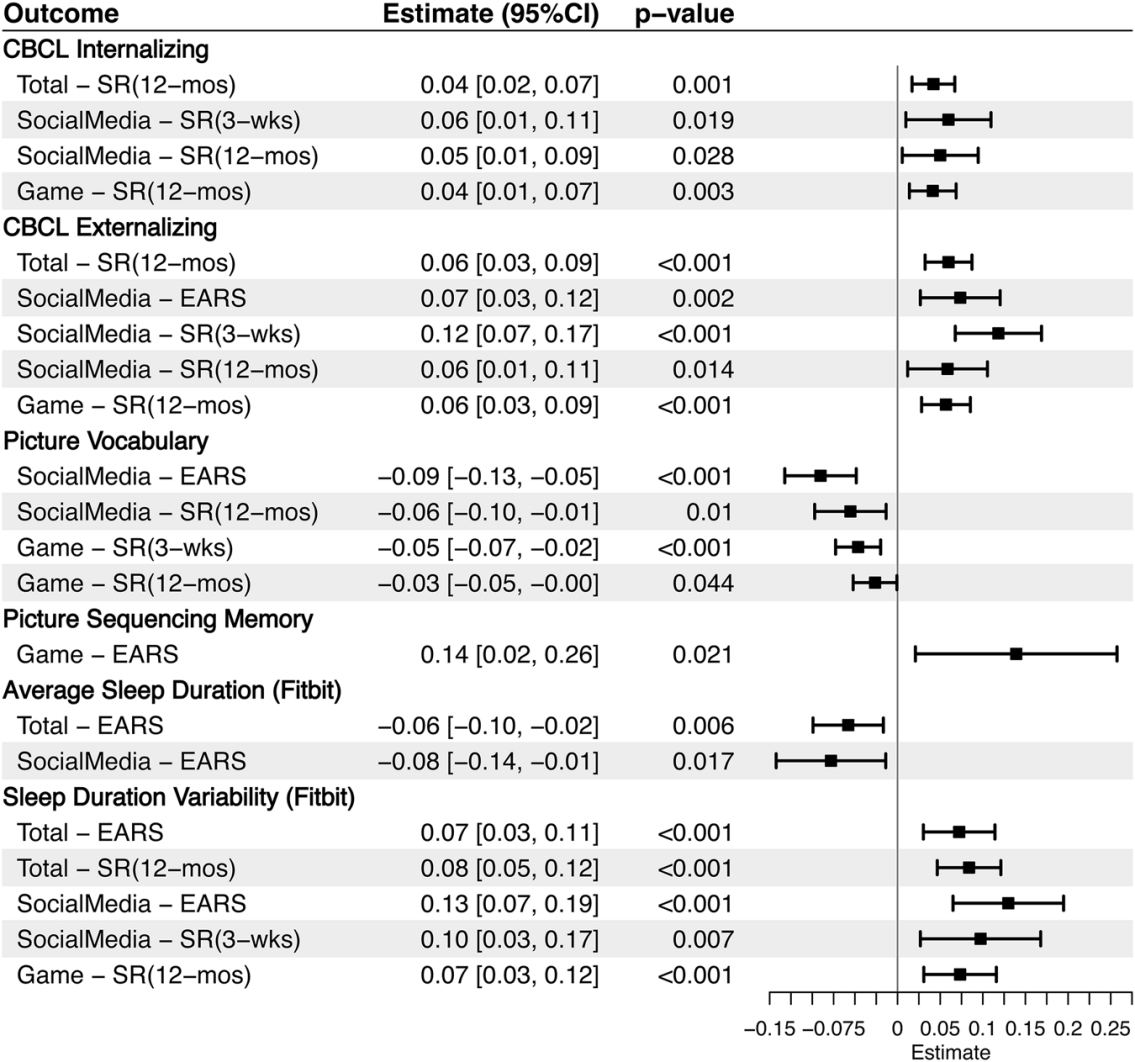
Forest plot of behavior-SMA relationship across objective and subjective social media measures. Here, SR(3-wks) stands for self-report screen time during post-sensing period and SR(12-mos) for self-report screen time in the past 12-months. EARS refers to the logged data collected by the effortless assessment of risk states app. Beta estimates were standardized. CI = confidence interval, CBCL = child behavior check list, SMAQ = social media addiction questionnaire, VGAQ = videogame addiction questionnaire.

Figure 3 presented the associations between social media use and behavioral problems and problematic technology usage using both objective and subjective metrics. Both objective and subjective measures of social media use were found to be related to externalizing behavior and SMAQ. Higher externalizing symptoms were related to greater social media use, as measured by both EARS (*β* = 0.07, 95% CI: 0.03–0.12) and self-report during the sensing period (*β* = 0.12, 95% CI: 0.07–0.17) and prior 12 months (*β* = 0.06, 95% CI: 0.01–0.11). Similarly, higher SMAQ were related to greater social media use, as measured by both EARS (*β* = 0.06, 95% CI: 0.01–0.11) and self-report during the sensing period (*β* = 0.08, 95% CI: 0.03–0.14) and prior 12 months (*β* = 0.15, 95% CI: 0.10–0.20).

Additionally, self-reported social media use during the sensing period (*β* = 0.06, 95% CI: 0.01–0.11) and over the prior 12 months (*β* = 0.05, 95% CI: 0.01–0.09) were positively associated with internalizing behaviors. Furthermore, self-reported social media use over the prior 12 months was also related to VGAQ (*β* = 0.05, 95% CI: 0.01–0.10).

## Discussion

This study leveraged intensive objectively logged smartphone app usage over a three-week period from the EARS passive tracking app, along with extensive self-reported technology use surveys in a sample of 495 adolescents. Several key findings emerged from our analyses. First, EARS-logged and post-sensing self-reported social media use were not significantly different, yet differences in past-year usage of social media were seen. Second, lower scores on a cognitive task (the picture vocabulary test) were associated with differences between EARS-logged and self-reported time. Third, both objective and subjective social media usage were robustly related to parent-reported externalizing problems and SMAQ scores. Collectively, findings highlight the value of both device-obtained and self-reported measures of SMA and suggest considering both types of measures in understanding youth mental health and functioning in the current digital technology environment.

Self-report data indicated that more than 94% of time spent on social media occurred on smartphones, suggesting that smartphones are the primary device for social media activity. The finding implies that social media use may be reliably captured by EARS-logged data. Consistent with previous research^14,15,45,46^, we observed moderate positive correlations between EARS-logged and self-reported use of social media. The mean differences in social media use between self-reported and EARS-logged measures were not significant for the 3-week sensing period but were significant when compared to 12-month retrospective reports. This suggests that adolescents can reasonably estimate use of social media over shorter recall periods.

Differences were observed related to gaming duration, with EARS significantly underestimating gaming time compared to self-report estimates over both the 3-week sensing and past-12-month recall periods. Only one-third of gaming time appeared to occur on smartphones, suggesting that adolescents often use various devices for gaming. The limitation of EARS in capturing gaming solely on smartphones likely generated this underestimation.

Interestingly, the association between picture vocabulary score and subjective-objective social media use difference suggests a potential cognitive influence on reporting accuracy. Self-reporting discrepancies may arise from memory recall biases, cognitive heuristics, and comprehension challenges, particularly in adolescents whose cognitive skills are still developing^2,47,48^. Answering self-report questions is often a cognitively demanding process, requiring individuals to comprehend questions, recall relevant behaviors, make inferences, and map responses onto available formats^49^. Even seemingly simple behavioral questions may introduce systematic biases, particularly if respondents struggle with comprehension or recall. This framework provides an important lens for understanding why individual differences in cognitive functioning involving vocabulary knowledge may influence self-reporting accuracy^49^. Additionally, self-reported cognitive difficulties in older adults at risk for Alzheimer’s disease suggest the importance of question interpretation and response biases in self-reports^50^. Specifically, vague item wording, incorrect assumptions about the consistency of cognitive difficulties across situations, and emotional responses were related to response biases (ibid). These findings align with research on self-reporting errors suggesting that both question structure and individual cognitive functioning relate to reporting accuracy. Similar mechanisms may contribute to reporting discrepancies in adolescents, as question comprehension, recall biases, and social desirability factors may interact with cognitive functioning to shape self-reported SMA estimates. Our findings suggest the importance of optimizing questionnaire design when assessing SMA in adolescents. Minor changes in question wording, format, or context may help improve recall accuracy, as previously demonstrated in studies of behavioral self-reports^49^.

While executive functioning (e.g., working memory, attentional control) is often implicated in recall accuracy^51–53^, less is known about the role of verbal cognitive skills, such as vocabulary knowledge, in self-reported SMA. Picture vocabulary, which measures verbal reasoning and comprehension^54^, may influence how adolescents interpret self-report questions and retrieve information from memory. Adolescents with lower vocabulary scores may struggle with question comprehension, leading to increased reliance on estimation heuristics rather than accurate recall. Given that self-awareness and self-monitoring require both executive and verbal cognitive skills^55^, further research with larger sample sizes may help better identify which cognitive domains contribute most to reporting discrepancies in SMA.

Notably, regardless of whether usage was objectively logged or subjectively reported, more frequent social media use was associated with higher externalizing behaviors and SMAQ scores, resonating with prior findings^56–59^. Objective measures are less susceptible to recall bias, potentially allowing for more precise effect-size estimations. Alternatively, subjective measures offer valuable insights into perceived impacts and contexts of social media use. For example, a positive association with internalizing behavior existed only for self-reported use. This relationship could potentially be influenced by subjective experiences of social media use, including feelings of comparison, inadequacy, or cyberbullying, which were not directly measurable through the employed objective metrics.

Previously, self-report measures have been reported as more robust predictors of problematic digital dependency symptoms than sensor-based durations^60^. Our prior work also found significant associations between self-report SMA and objectively measured brain features^11,61^ that were previously linked to early initiation of alcohol use^62^. Therefore, from a measurement perspective, although passive sensing methods may offer a more ecologically valid assessment of SMA by capturing real-time engagement patterns and minimizing recall biases associated with self-reports^63^, self-reports are clinically relevant and may potentially capture individual perceptions and contextual factors related to SMA. Integrating both objective and subjective measures may promote a more comprehensive understanding of relationships between social media use and behavioral outcomes.

While this investigation focuses on screen time, challenges of self-report accuracy extend to other behaviors commonly assessed through self-reports, such as sleep patterns and physical activity^64,65^. As demonstrated in sleep research, subjective and objective measures may capture distinct aspects of the same behavior, such as sleep duration^66^, which could lead to systematic discrepancies in reported outcomes. These findings underscore the importance of methodological integration, as different measurement approaches may influence research conclusions. Recommendations to parents regarding passive monitoring of youth SMA, sleep, exercise, and possibly other domains could support personalized discussions on healthy versus risky behaviors and inform health recommendations from relevant academic/clinical groups and policymakers.

Finally, we note that our study has several limitations. First, the relatively small sample size and narrow age range in this study limit our ability to fully examine how developmental stage influences reporting accuracy. It is possible that adolescents’ cognitive and social maturity may influence the accuracy of self-reported SMA^26^. Younger adolescents may struggle with estimating their screen time due to underdeveloped metacognitive abilities and time perception^67,68^, whereas older adolescents may provide more accurate estimates but remain susceptible to social desirability bias^69,70^. Additionally, shifting attention spans and the dynamic nature of digital media engagement may further complicate recall accuracy. Prior research suggests that self-reporting errors increase when SMA involves multitasking across multiple digital platforms^71,72^. Given these challenges, future studies should further investigate developmental variations in self-report reliability and consider integrating passive sensing methodologies to complement self-reported data. Second, this investigation did not examine associations between reporting bias and other cognitive functions, such as executive functioning and attentional control. The Flanker Test, which assesses executive functioning and tendencies to inhibit attention to irrelevant information, and the card sorting task, which measures set-shifting tendencies may be more directly relevant to reporting biases^73^. However, these measures were excluded due to high missingness. Thus, our findings should be interpreted with caution, and future studies with larger samples should explore these potential relationships. Third, EARS-logged data were based on youth with Android phones, who were more likely boys and from families with lower social economic status, as seen in prior studies^74^. Therefore, data from both Android and iOS sources should be examined for replicability and extension of the current findings. Additionally, EARS-logged data were based on foreground usage, which may potentially miss multitasking behaviors or background activities like music playing. This leaves uncertainty about the proportion of time spent multitasking. Fourth, the 3-week tracking period may not fully represent participants’ typical screen time behavior, as SMA patterns may vary across different contexts (e.g., school breaks or specific life events). Additionally, single-device tracking may introduce systematic biases in SMA research, as adolescents frequently engage with multiple devices, including smartphones, tablets, computers, televisions, and gaming consoles^75^. Therefore, tracking screen time, especially gaming, from a single device may fail to capture the full scope of their media engagement^26^. Fifth, recent research highlights the limitations of relying solely on raw screen time metrics to assess addictive behaviors^76^. Merely measuring screen time may overlook the complexity of addiction in digital environments. For example, research suggests that use duration and frequency capture distinct behavioral patterns^29^. Time spent using one’s smartphone, rather than frequency of phone checking, was a stronger predictor of problematic smartphone use, while individuals with higher proneness to negative affect, specifically depression, were less likely to engage in frequent phone-checking behaviors (ibid). Aspects of media use, including social comparison and exposure to harassment, correlate more closely with mental health outcomes than screen time duration^77^. These findings suggest the importance of considering both quantity and context of digital interactions for understanding potential impacts on psychological well-being^78^. Future research should prioritize comprehensive assessment tools that capture multiple dimensions of addictive screen use. Additionally, studies should explore advanced multi-device tracking technologies while incorporating demographic factors to ensure that measurement strategies accurately reflect the complexity of adolescent screen engagement.

In summary, our results suggest that agreement between subjective and objective metrics relate to digital activity type and recall timeframe. Adolescents may demonstrate more reliable self-reporting of shorter-term social media use. However, accurately measuring gaming via EARS poses challenges possibly due to its restriction to smartphone usage. Incorporating objective data from multiple platforms regularly used by youth is important for gaining clarity regarding actual usage and potential impacts. Studies have suggested that looking solely at total durations, while relevant, may provide somewhat limited value^13^. Evaluating patterns of usage, contextual factors, and fluctuations in engagement may reveal richer insights^3^.

## Supplementary information

Supplementary information

## Data Availability

The datasets analyzed during the current study are available in the ABCD Data repository (https://nda.nih.gov/abcd). Researchers with an approved NDA Data Use Certification (DUC) may obtain ABCD study data.

## Code availability

The code for this study can be found at https://github.com/ZhaoCUMC/ABCD_EARS.
