# Supplementary material for: Examining measurement discrepancies in adolescent screen media activity with insights from the ABCD study
Source: Npj Ment Health Res. 2025 May 10;4:15. doi: 10.1038/s44184-025-00131-z (PMC12064680; doi:10.1038/s44184-025-00131-z)
Supplement: Supplementary file 1 — Supplementary information [file 44184_2025_131_MOESM1_ESM.pdf]

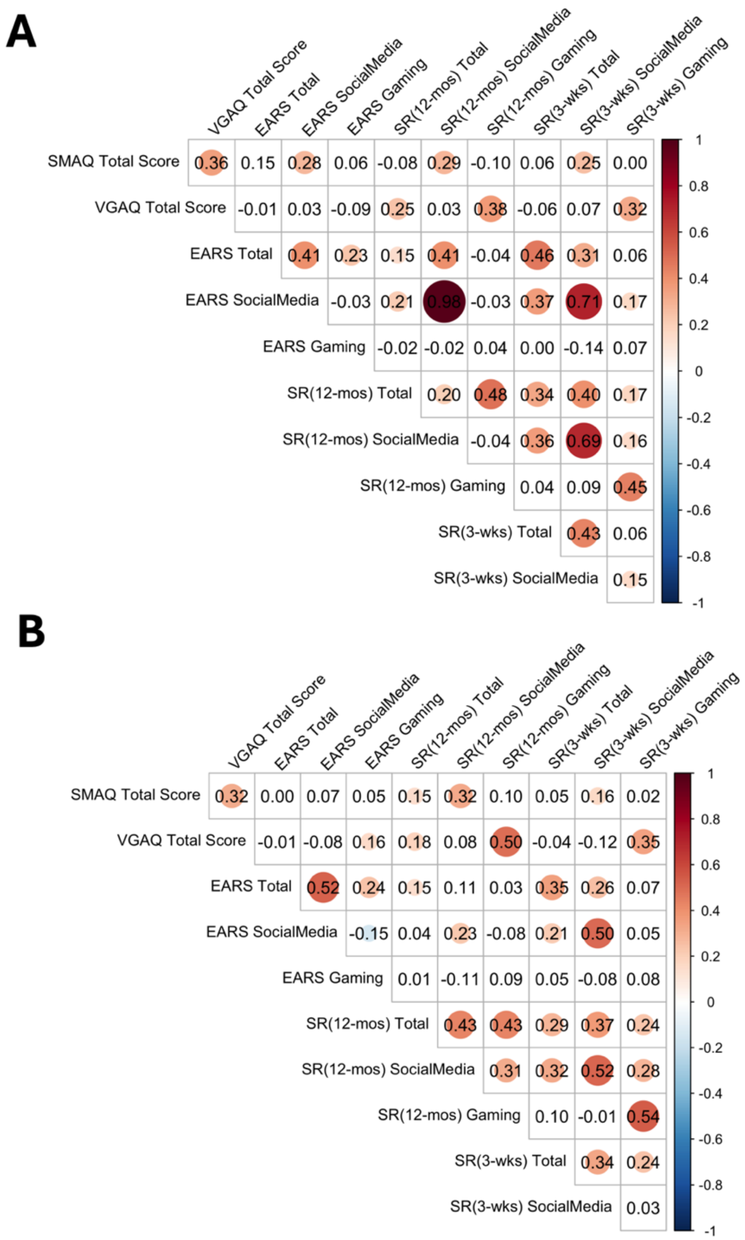

Supplementary Figure 1. Pairwise correlations among key SMA measures including self-reported screen time, EARS-related measures, and addiction scores are shown for (A) accurate and (B) biased-reporting groups. Each cell represents a correlation coefficient, with significant correlations highlighted by circles, where size and color indicate strength and direction. The accurate group includes participants with an objective-subjective discrepancy of ⩽30 minutes, while the biased-reporting group includes those with a discrepancy >30 minutes

Supplementary Table 1. Key social media and game apps used by participants with EARS app usage data[1]

Social Media		Video Game	
App Title	No. Users	App Title	No. Users
TikTok	279 (56.4%)	Google Play Games	134 (27.1%)
Instagram	256 (51.7%)	Roblox	116 (23.4%)
Snapchat	228 (46.1%)	Among Us	87 (17.6%)
Facebook	105 (21.2%)	Pokémon GO	65 (13.1%)
Reddit	95 (19.2%)	Clash Royale	63 (12.7%)
Twitter	92 (18.6%)	Xbox	58 (11.7%)
sendit	18 (3.6%)	Minecraft	54 (10.9%)
Tumblr	16 (3.2%)	Clash of Clans	52 (10.5%)
BAND	11 (2.2%)	Subway Surfers	47 (9.5%)
		BitLife	34 (6.9%)
		PlayStation App	29 (5.9%)
		Geometry Dash Lite	27 (5.5%)
		Magic Tiles 3	23 (4.7%)
		Genshin Impact	21 (4.2%)
		Brawl Stars	21 (4.2%)
		Call of Duty Mobile Season 6	20 (4.0%)
		Meta Quest	20 (4.0%)
		Gacha Club	18 (3.6%)
		Egg, Inc.	18 (3.6%)
		Cookie Run: Kingdom	17 (3.4%)
		Steam	16 (3.2%)
		Bloons TD 6	15 (3.0%)
		Solar Smash	14 (2.8%)
		State.io	13 (2.6%)
		Tomb of the Mask	12 (2.4%)
		Geometry Dash	12 (2.4%)
		High Heels!	12 (2.4%)
		WorldBox	11 (2.2%)
		Retro Bowl	11 (2.2%)
		Paint by Number: Coloring Game	11 (2.2%)
		Plants vs. Zombies	10 (2.0%)
		Nonogram.com	10 (2.0%)
		Episode	10 (2.0%)
		Jetpack Joyride	10 (2.0%)
		MISTPLAY	10 (2.0%)
		Obey Me!	10 (2.0%)
		Crossy Road	10 (2.02%)
		Dragon City Mobile	10 (2.02%)

[1]Key apps were defined as apps accessed by at least 10 individuals during the 3-week sensing period. EARS refers to the Effortless Assessment of Risk States app.

Supplementary Table 2: Subjective Measures

	Data Source	Measures on a typical day	Original measures used for data processing	Original Levels	Recode to Continuous Measures (Hrs)
SR[1] (3-wks)	EARS - Post-Assessment Survey (nt_y_ears_qtn)	Total	ears_post_6_y (wkdy); ears_post_time_7_y (wknd)	0 = 0 | 1 = 30 minutes | 2 = 45 minutes | 3 = 1 hour | 4 = 1.5 hours | 5 = 2 hours | 6 = 2.5 hours | 7 = 3 hours | 8 = 4 hours | 9 = 5 hours | 10 = 6 hours | 11 = 7 hours | 12 = 8 hours | 13 = 9 hours | 14 = 10 hours | 15 = 11 hours | 16 = 12 hours | 17 = 13 hours | 18 = 14 hours | 19 = 15 hours | 20 = 16 hours | 21 = 17 hours | 22 = 18 hours | 23 = 19 hours | 24 = 20 hours | 25 = 21 hours | 26 = 22 hours | 27 = 23 hours | 28 = 24 hours | 999 = Don't know	0, 0.5, 0.75, 1, 1,5, 2, 2.5, 3-24
		Social Media	ears_post_3_y (wkdy); ears_post_time_4_y (wknd)		
		Gaming	single-player video games: ears_post_y (wkdy); ears_post_time_1_y (wknd) multiplayer video games: ears_post_1_y (wkdy); ears_post_time_2_y (wknd)		
SR[1] (12-mos)	Screen Time Questionnaire (nt_y_st)	Total	screentime_wkdy_typical_hr + screentime_wkdy_typical_min (wkdy); screentime_wknd_typical_hr + screentime_wknd_t_min (wknd)	hr: 0=0; 1=1; 2=2; 3=3; 4=4; 5=5; 6=6; 7=7; 8=8; 9=9; 10=10; 11=11; 12=12; 13=13; 14=14; 15=15; 16=16; 17=17; 18=18; 19=19; 20=20; 21=21; 22=22; 23=23 min: 0=0; 15=15; 30=30; 45=45	0, 0.25, 0.5, 0.75, 1, 1.25, 1.5, 1.75, …, 23.75
		Social Media	screentime_6_wkdy_hr + screentime_6_wkdy_min (wkdy); screentime_7_wkdy_hr + screentime_7_wkdy_min (wknd)		
		Gaming	single-player video games: screentime_3_wkdy_hr + screentime_3_wkdy_min (wkdy); screentime_9_wknd_hr + screentime_9_wknd_min (wknd) multiplayer video games: screentime_4_wkdy_hr + screentime_4_wkdy_min (wkdy); screentime_10_wknd_hr + screentime_10_wknd_min (wknd)		

[1]SR=self-report; SR (3-wks) means post-sensing self-report data; SR (12-mos) means past-year self-report data

Supplementary Table 3. Top 20 apps ranked by average daily app usage per participant

App Title	Average Daily App Usage across all 495 participants	Average Daily App Usage across participants with nonzero app usage[1]	No. Users (%)	Categories[2]
YouTube	64.57	72.31	442 (89.29%)	Video Players
TikTok	60.33	107.03	279 (56.36%)	Social
Snapchat	17.79	38.62	228 (46.06%)	Social
Instagram	16.95	32.77	256 (51.72%)	Social
Google Chrome	14.07	14.60	477 (96.36%)	Communication
Discord	9.29	18.63	247 (49.9%)	Communication
Netflix	7.49	21.94	169 (34.14%)	Video Players
Wattpad	4.19	53.14	39 (7.88%)	Books & Reference
Samsung One UI Home	3.60	7.05	253 (51.11%)	Personalization
Clock	2.92	4.45	325 (65.66%)	Tools
Google	2.77	3.02	455 (91.92%)	Tools
WEBTOON	2.42	16.60	72 (14.55%)	Comics
Twitch	2.30	9.98	114 (23.03%)	Video Players
YouTube Music	2.29	10.78	105 (21.21%)	Music & Audio
Samsung Internet Browser	2.24	10.09	110 (22.22%)	Communication
Spotify	2.23	4.14	267 (53.94%)	Music & Audio
Hulu	2.19	20.04	54 (10.91%)	Video Players
Reddit	2.17	11.32	95 (19.19%)	Social
Minecraft	2.02	18.48	54 (10.91%)	Game
Disney+	1.97	10.49	93 (18.79%)	Video Players

[1]Average daily app usage for each app was calculated as total app usage divided by the number of individuals who had nonzero app usage data for that app during the 3-week passive sensing period.

[2]App categories were defined by Google Play except that Snapchat was defined as social media apps in this investigation.

Supplementary Table 4a. Multinomial logistic regression models testing associations between NIH Toolbox Picture Sequence Memory and accurate- and over-reporting of social media use

	Cutoff=0.5hr		Cutoff=1hr	
	Accurate: 222; over: 105		Accurate: 289; over: 69	
	OR [95%CI]	p-value	OR [95%CI]	p-value
Picture Sequence Memory	0.99 [0.98-1.01]	0.332	1.00 [0.98-1.02]	0.951
Age (yrs)	1.18 [0.83-1.68]	0.358	1.15 [0.78-1.71]	0.486
Sex at Birth (Ref: Male)				
Female	1.14 [0.69-1.89]	0.606	1.08 [0.62-1.90]	0.782
Race Ethnicity (Ref: White)				
Black	3.60 [1.36-9.54]	0.010	3.47 [1.35-8.91]	0.010
Hispanic	0.96 [0.47-1.94]	0.904	1.39 [0.63-3.05]	0.412
Other	0.62 [0.28-1.38]	0.242	0.82 [0.33-2.00]	0.660
Family Income (US$) (Ref: [>100k])				
[50-100k]	0.71 [0.37-1.34]	0.292	0.70 [0.33-1.46]	0.340
[<50k]	0.75 [0.32-1.78]	0.517	0.70 [0.27-1.81]	0.456
Parental Education (Ref: Post Graduate Degree)				
Bachelor	1.11 [0.59-2.10]	0.747	0.74 [0.35-1.55]	0.420
Some College	1.41 [0.67-2.99]	0.370	1.17 [0.52-2.65]	0.708
High School or Below	1.54 [0.59-4.06]	0.380	1.13 [0.39-3.25]	0.827
Parental Marital (Ref: Married)				
Not Married	2.24 [1.25-4.02]	0.007	1.63 [0.85-3.13]	0.141

Supplementary Table 4b. Multinomial logistic regression models testing associations between NIH Toolbox Picture Sequence Memory and accurate- and under-reporting of social media use

	Cutoff=0.5hr		Cutoff=1hr	
	Accurate: 222; under: 144		Accurate: 289; under: 113	
	OR [95%CI]	p-value	OR [95%CI]	p-value
Picture Sequence Memory	0.99 [0.98-1.01]	0.477	1.00 [0.98-1.01]	0.708
Age (yrs)	1.31 [0.95-1.80]	0.098	1.32 [0.95-1.83]	0.098
Sex at Birth (Ref: Male)				
Female	1.21 [0.77-1.89]	0.407	1.15 [0.73-1.83]	0.541
Race Ethnicity (Ref: White)				
Black	2.64 [1.03-6.79]	0.043	1.83 [0.78-4.30]	0.166
Hispanic	1.42 [0.79-2.58]	0.245	1.80 [0.99-3.27]	0.052
Other	0.76 [0.38-1.51]	0.431	0.74 [0.34-1.59]	0.434
Family Income (US$) (Ref: [>100k])				
[50-100k]	0.68 [0.38-1.21]	0.191	0.91 [0.50-1.67]	0.771
[<50k]	1.06 [0.50-2.24]	0.880	1.13 [0.52-2.43]	0.754
Parental Education (Ref: Post Graduate Degree)				
Bachelor	1.20 [0.67-2.17]	0.542	1.20 [0.64-2.25]	0.566
Some College	1.99 [1.01-3.91]	0.047	1.61 [0.79-3.27]	0.186
High School or Below	1.62 [0.66-3.93]	0.291	1.66 [0.68-4.03]	0.265
Parental Marital (Ref: Married)				
Not Married	1.51 [0.88-2.60]	0.134	1.12 [0.65-1.93]	0.682

Supplementary Table 5a. Multinomial logistic regression models testing associations between NIH Toolbox List Sorting Working Memory and accurate- and over-reporting of social media use

	Cutoff=0.5hr		Cutoff=1hr	
	Accurate: 221; over: 105		Accurate: 288; over: 69	
	OR [95%CI]	p-value	OR [95%CI]	p-value
List Sorting Working Memory	0.98 [0.96-1.00]	0.028	0.99 [0.96-1.01]	0.164
Age (yrs)	1.21 [0.85-1.73]	0.294	1.18 [0.79-1.76]	0.421
Sex at Birth (Ref: Male)				
Female	1.13 [0.68-1.86]	0.644	1.10 [0.63-1.93]	0.733
Race Ethnicity (Ref: White)				
Black	3.09 [1.16-8.25]	0.024	2.96 [1.14-7.66]	0.026
Hispanic	0.91 [0.45-1.84]	0.796	1.35 [0.62-2.96]	0.447
Other	0.63 [0.28-1.38]	0.249	0.80 [0.33-1.96]	0.628
Family Income (US$) (Ref: [>100k])				
[50-100k]	0.75 [0.39-1.42]	0.372	0.72 [0.35-1.51]	0.389
[<50k]	0.77 [0.32-1.81]	0.542	0.70 [0.27-1.82]	0.465
Parental Education (Ref: Post Graduate Degree)				
Bachelor	1.01 [0.53-1.93]	0.972	0.68 [0.32-1.43]	0.305
Some College	1.26 [0.59-2.70]	0.548	1.05 [0.46-2.40]	0.905
High School or Below	1.35 [0.51-3.58]	0.547	1.00 [0.34-2.90]	0.995
Parental Marital (Ref: Married)				
Not Married	2.36 [1.31-4.26]	0.004	1.67 [0.87-3.21]	0.123

Supplementary Table 5b. Multinomial logistic regression models testing associations between NIH Toolbox List Sorting Working Memory and accurate- and under-reporting of social media use

	Cutoff=0.5hr		Cutoff=1hr	
	Accurate: 221; under: 145		Accurate: 288; under: 114	
	OR [95%CI]	p-value	OR [95%CI]	p-value
List Sorting Working Memory	0.99 [0.97-1.01]	0.323	1.00 [0.98-1.02]	0.892
Age (yrs)	1.33 [0.97-1.83]	0.081	1.33 [0.96-1.85]	0.088
Sex at Birth (Ref: Male)				
Female	1.17 [0.75-1.83]	0.483	1.13 [0.71-1.78]	0.611
Race Ethnicity (Ref: White)				
Black	2.47 [0.96-6.37]	0.061	1.80 [0.76-4.27]	0.179
Hispanic	1.34 [0.74-2.43]	0.332	1.73 [0.96-3.14]	0.070
Other	0.76 [0.38-1.50]	0.425	0.73 [0.34-1.57]	0.423
Family Income (US$) (Ref: [>100k])				
[50-100k]	0.71 [0.40-1.27]	0.251	0.95 [0.52-1.73]	0.865
[<50k]	1.06 [0.50-2.24]	0.886	1.13 [0.52-2.43]	0.762
Parental Education (Ref: Post Graduate Degree)				
Bachelor	1.16 [0.64-2.10]	0.624	1.20 [0.64-2.25]	0.576
Some College	1.93 [0.98-3.82]	0.059	1.62 [0.80-3.31]	0.182
High School or Below	1.66 [0.69-4.03]	0.261	1.78 [0.74-4.33]	0.199
Parental Marital (Ref: Married)				
Not Married	1.54 [0.90-2.66]	0.117	1.11 [0.65-1.93]	0.696

Supplementary Table 6a. Multinomial logistic regression models testing associations between NIH Toolbox Oral Reading Recognition and accurate- and over-reporting of social media use

	Cutoff=0.5hr		Cutoff=1hr	
	Accurate: 221; over: 106		Accurate: 289; over: 69	
	OR [95%CI]	p-value	OR [95%CI]	p-value
Oral Reading Recognition	0.98 [0.95-1.02]	0.354	0.98 [0.94-1.02]	0.246
Age (yrs)	1.21 [0.85-1.73]	0.278	1.18 [0.80-1.76]	0.405
Sex at Birth (Ref: Male)				
Female	1.11 [0.68-1.83]	0.676	1.11 [0.64-1.95]	0.710
Race Ethnicity (Ref: White)				
Black	3.47 [1.30-9.27]	0.013	3.03 [1.17-7.87]	0.022
Hispanic	0.91 [0.45-1.84]	0.789	1.36 [0.62-2.97]	0.449
Other	0.64 [0.29-1.41]	0.271	0.83 [0.34-2.04]	0.686
Family Income (US$) (Ref: [>100k])				
[50-100k]	0.77 [0.41-1.46]	0.425	0.71 [0.34-1.50]	0.370
[<50k]	0.79 [0.34-1.87]	0.598	0.70 [0.27-1.84]	0.471
Parental Education (Ref: Post Graduate Degree)				
Bachelor	1.14 [0.61-2.16]	0.678	0.72 [0.34-1.50]	0.375
Some College	1.36 [0.64-2.88]	0.429	1.08 [0.47-2.47]	0.851
High School or Below	1.49 [0.56-3.92]	0.423	1.01 [0.34-2.97]	0.987
Parental Marital (Ref: Married)				
Not Married	2.27 [1.26-4.09]	0.006	1.69 [0.88-3.27]	0.116

Supplementary Table 6b. Multinomial logistic regression models testing associations between NIH Toolbox Oral Reading Recognition and accurate- and under-reporting of social media use

	Cutoff=0.5hr		Cutoff=1hr	
	Accurate: 221; under: 145		Accurate: 289; under: 114	
	OR [95%CI]	p-value	OR [95%CI]	p-value
Oral Reading Recognition	0.98 [0.94-1.01]	0.152	0.97 [0.94-1.01]	0.154
Age (yrs)	1.36 [0.99-1.87]	0.059	1.37 [0.99-1.90]	0.061
Sex at Birth (Ref: Male)				
Female	1.20 [0.77-1.88]	0.426	1.15 [0.73-1.82]	0.543
Race Ethnicity (Ref: White)				
Black	2.39 [0.92-6.19]	0.072	1.61 [0.68-3.83]	0.279
Hispanic	1.33 [0.73-2.41]	0.347	1.70 [0.94-3.08]	0.081
Other	0.78 [0.39-1.54]	0.469	0.75 [0.35-1.60]	0.452
Family Income (US$) (Ref: [>100k])				
[50-100k]	0.72 [0.40-1.28]	0.265	0.95 [0.52-1.73]	0.870
[<50k]	1.07 [0.51-2.28]	0.854	1.13 [0.52-2.43]	0.763
Parental Education (Ref: Post Graduate Degree)				
Bachelor	1.19 [0.66-2.16]	0.557	1.18 [0.63-2.20]	0.613
Some College	1.89 [0.96-3.74]	0.066	1.53 [0.75-3.12]	0.242
High School or Below	1.60 [0.66-3.90]	0.300	1.63 [0.67-3.98]	0.284
Parental Marital (Ref: Married)				
Not Married	1.55 [0.90-2.67]	0.115	1.15 [0.66-1.99]	0.619

Supplementary Table 7a. Multinomial logistic regression models testing associations between NIH Toolbox Picture Vocabulary and accurate- and over-reporting of social media use with pubertal development stages as covariate

	Cutoff=0.5hr		Cutoff=1hr	
	Accurate: 221; over: 105		Accurate: 289; over: 68	
	OR [95%CI]	p-value	OR [95%CI]	p-value
Picture Vocabulary	0.97 [0.94-1.01]	0.114	0.97 [0.94-1.01]	0.173
Age (yrs)	1.16 [0.80-1.68]	0.426	1.18 [0.78-1.79]	0.433
Sex at Birth (Ref: Male)				
Female	0.95 [0.54-1.68]	0.866	1.00 [0.53-1.89]	0.992
Race Ethnicity (Ref: White)				
Black	3.22 [1.19-8.73]	0.021	3.02 [1.14-8.03]	0.026
Hispanic	0.84 [0.41-1.73]	0.638	1.33 [0.60-2.96]	0.484
Other	0.63 [0.29-1.40]	0.258	0.81 [0.33-2.00]	0.648
Family Income (US$) (Ref: [>100k])				
[50-100k]	0.66 [0.34-1.25]	0.202	0.60 [0.28-1.27]	0.181
[<50k]	0.68 [0.29-1.63]	0.392	0.60 [0.23-1.59]	0.309
Parental Education (Ref: Post Graduate Degree)				
Bachelor	1.15 [0.60-2.20]	0.664	0.67 [0.32-1.43]	0.305
Some College	1.29 [0.60-2.80]	0.514	1.09 [0.47-2.52]	0.843
High School or Below	1.54 [0.57-4.15]	0.394	1.02 [0.34-3.03]	0.975
Parental Marital (Ref: Married)				
Not Married	2.33 [1.29-4.21]	0.005	1.71 [0.88-3.29]	0.111
Pubertal Development (Ref: Late or Post Puberty)				
Mid Puberty	0.90 [0.49-1.64]	0.722	0.79 [0.40-1.56]	0.492
Pre- or Early Puberty	0.27 [0.08-0.93]	0.038	0.63 [0.18-2.15]	0.459

Supplementary Table 7b. Multinomial logistic regression models testing associations between NIH Toolbox Picture Vocabulary and accurate- and under-reporting of social media use with pubertal development stages as covariate

	Cutoff=0.5hr		Cutoff=1hr	
	Accurate: 221; under: 144		Accurate: 289; under: 113	
	OR [95%CI]	p-value	OR [95%CI]	p-value
Picture Vocabulary	0.96 [0.93-0.99]	0.007	0.96 [0.93-0.99]	0.007
Age (yrs)	1.42 [1.02-1.98]	0.039	1.52 [1.08-2.15]	0.017
Sex at Birth (Ref: Male)				
Female	1.14 [0.68-1.92]	0.625	1.30 [0.76-2.24]	0.340
Race Ethnicity (Ref: White)				
Black	2.07 [0.79-5.44]	0.141	1.36 [0.56-3.31]	0.498
Hispanic	1.25 [0.68-2.30]	0.465	1.64 [0.89-3.02]	0.112
Other	0.75 [0.37-1.50]	0.410	0.72 [0.33-1.56]	0.406
Family Income (US$) (Ref: [>100k])				
[50-100k]	0.64 [0.36-1.16]	0.143	0.91 [0.49-1.68]	0.759
[<50k]	0.98 [0.46-2.10]	0.962	1.09 [0.50-2.37]	0.832
Parental Education (Ref: Post Graduate Degree)				
Bachelor	1.15 [0.63-2.10]	0.643	1.10 [0.58-2.07]	0.775
Some College	1.70 [0.85-3.41]	0.131	1.35 [0.66-2.79]	0.412
High School or Below	1.30 [0.52-3.26]	0.571	1.25 [0.50-3.15]	0.632
Parental Marital (Ref: Married)				
Not Married	1.60 [0.93-2.77]	0.092	1.18 [0.68-2.05]	0.558
Pubertal Development (Ref: Late or Post Puberty)				
Mid Puberty	0.90 [0.51-1.58]	0.712	1.13 [0.64-2.02]	0.673
Pre- or Early Puberty	0.88 [0.37-2.10]	0.773	1.73 [0.71-4.20]	0.227
